# Case report: A case study of variant calling pipeline selection effect on the molecular diagnostics outcome

**DOI:** 10.3389/fonc.2024.1422811

**Published:** 2024-10-31

**Authors:** Rostislav Skitchenko, Sergey Smirnov, Mikhail Krapivin, Anna Smirnova, Mykyta Artomov, Alexander Loboda, Yulia Dinikina

**Affiliations:** ^1^ Laboratory of Computer Modelling and Artificial Intelligence, Almazov National Medical Research Centre, St. Petersburg, Russia; ^2^ Computer Technologies Laboratory, National Research University of Information Technologies, Mechanics and Optics, St. Petersburg, Russia; ^3^ The Institute for Genomic Medicine, Nationwide Children’s Hospital, Columbus, OH, United States; ^4^ Department of Pediatrics, The Ohio State University, Columbus, OH, United States

**Keywords:** SHH medulloblastoma, pediatric oncology, germline, DeepVariant, variant calling, HaplotypeCaller, undiagnosed cases

## Abstract

Next-generation sequencing technologies have not only defined a breakthrough in medical genetics, but also been able to enter routine clinical practice to determine individual genetic susceptibilities. Modern technological developments are routinely introduced to genetic analysis overtaking the established approaches, potentially raising a number of challenges. To what extent is the advantage of new methodologies in synthetic metrics, such as precision and recall, more important than stability and reproducibility? Could differences in the technical protocol for calling variants be crucial to the diagnosis and, by extension, the patient’s treatment strategy? A regulatory review process may delay the incorporation of potentially beneficial technologies, resulting in missed opportunities to make the right medical decisions. On the other hand, a blind adoption of new technologies based solely on synthetic metrics of precision and recall can lead to incorrect conclusions and adverse outcomes for the specific patient. Here, we use the example of a patient with a WHO-diagnosed desmoplastic/nodular SHH-medulloblastoma to explore how the choice of DNA variant search protocol affects the genetic diagnostics outcome.

## Introduction

Medulloblastoma (MB) is one of the most widespread malignant brain tumors in children (WHO Class IV). It accounts for approximately 62.4% of embryonal tumors, making it the most common type within this category (1). The annual incidence of MB in children is estimated to be approximately 5 cases per 1 million children (1).

Histomorphologically, MB is characterized as an embryonal tumor originating in the cerebellum and is believed to arise from distinct neural stem or progenitor cells during early developmental stages (2). MB encompasses four molecularly distinct subgroups: *wingless-type* (WNT), *Sonic Hedgehog* (SHH), Group 3, and Group 4 (3). SHH-type MB comprises approximately 30% of all MB cases and primarily affects patients below 3 years of age and those above 16 years, with a lower incidence observed in the 3- to 16-year age group (4). Survival outcomes for SHH-type MB are highly influenced by specific subtypes within the SHH subgroup (5).

The SHH signaling pathway plays a pivotal role in tissue and organ development, performing essential functions in morphogenesis, mitogenesis, and cellular differentiation processes (6). It is obvious that genetic variants in crucial genes of the SHH pathway, such as *Protein patched homolog 1* (*PTCH1*), *2* (*PTCH2*), *SUFU Negative Regulator Of Hedgehog Signaling* (*SUFU*), and *Smoothened, frizzled class receptor* (*SMO*), as well as in related transcription factors such as *Family Zinc Finger 1* (*GLI1*) and *2* (*GLI2*), directly influence MB pathogenesis. The vast majority of MB cases are explained by somatic mutations and epigenetic modifications. However, for 54% of cases, the molecular genetics profile of MB could not be established (7). The critical impact of germline and somatic mutations in *TP53* on the prognosis of survival in SHH-MB has likewise long been known (8). Recently putative causal germline mutations have been reported in MB patients, for example, germline mutations in *PTEN*, *ELP1*, and *PHOX2B* genes (9–11). Similar effects of germline variants have been observed in pediatric MB cases, e.g., in *MSH2*, *RAD50*, and *NBN* genes (12).

Here, using the genetic data from a pediatric patient with WHO-diagnosed desmoplastic/nodular SHH-MB, we highlight how the choice of variant calling platform affects the results of molecular diagnosis. Resolution of the issues highlighted in this study could potentially reduce the number of undiagnosed MB.

## Patient presentation and methods

### Patient’s health track

The infant patient was born with multiple intellectual and developmental disabilities, including cleft of the hard and soft palate of the upper lip and alveolar process on the right, as well as anomalies of the eyeball development: microphthalmos, microcornea, and anterior colobomatous cyst of the orbit.

**Figure 1:**
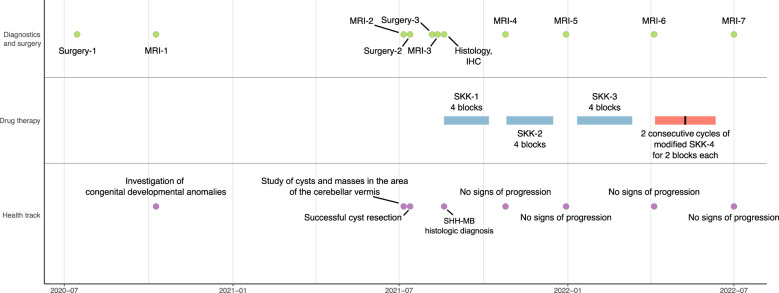
Clinical timeline of the patient.

The health and treatment track included several surgeries and monitoring of the recovery dynamics by MRI (**Figure 1**). Prior to the onset of oncological symptoms, the patient underwent cleft lip and palate repair in the summer of 2020. The first MRI in the fall of 2020 showed dysgenesis of the corpus callosum, dilation of the subarachnoid liquor spaces, ventricular system, and right orbital abnormality—cystic eye, presumably congenital.

The second MRI dated in the summer of 2021 was diagnostic for MB (see the Diagnostics section). Microsurgical removal of the cerebellar vermis and IV ventricle tumor was performed later that summer using intraoperative navigation. Findings of the histological and immunohistochemical analysis were consistent with SHH-MB. Regarding family history, no inherited syndromes associated with MB were identified.

Based on the post-surgery third MRI data of the brain and spinal cord, the patient disease stage was classified as R0M0, according to the histological results following the HIT-SKK 2017 protocol [version 4.0, 2017; Therapieprotokoll für Säuglinge und Kleinkinder (SKK) mit Hirntumoren (Brain Tumor Radiotherapy for Infants and Toddlers with Medulloblastoma)] (13, 14). The patient was recommended for polychemotherapy for MB in the 0- to 5-year-old age group, with classification M0 under the SKK scheme (13). According to the protocol, the chemotherapy plan included three consequential cycles of regular SKK and two cycles of modified SKK. After the first SKK, the MRI analysis showed positive dynamics: reduction of signs of pathologic contrast agent accumulation in the periphery of the postoperative cavity. Taking into account the data of the MRI, continuation of chemotherapy (second block of SKK) was prescribed. No further signs of cancer progression were identified. At the time of the last contact with the patient in April 2024, there was no evidence of recurrence, suggesting remission for at least 32 months.

### Diagnostics

**Figure 2:**
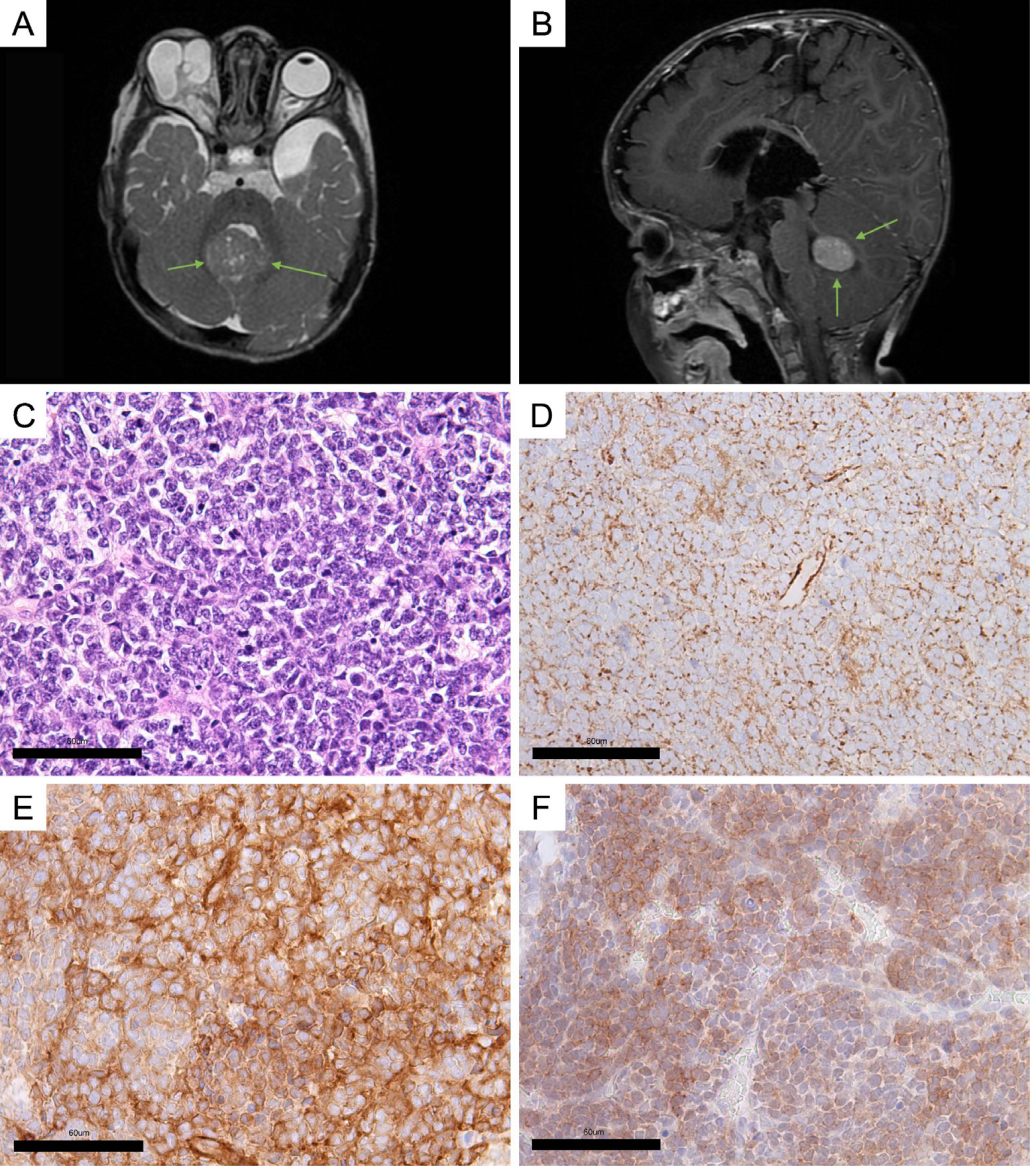
Clinical and histological characteristics. **(A, B)** MRI screens of the brain (green arrows indicate a cerebellar vermis tumor). **(A)** Axial plane; **(B)** sagittal plane. **(C)** Hematoxylin–eosin staining of the MB sample. **(D–F)** Immunohistochemical (IHC) staining of tumor sample: **(D)** beta-catenin; **(E)** filamin A; **(F)** GAB1. The scales were manually added to the figures.

Baseline MRI showed a left temporal lobe arachnoid cyst and a mass in the vermis of the cerebellum on the right side (**Figures 2A, B**).

Hematoxylin–eosin staining analysis was used for histomorphological classification of MB. The analysis revealed a large number of cells at the stage of mitotic division, indicating rapid tumor growth (**Figure 2C**). The tumor was assigned a Grade 4 status. Based on characteristic features such as nodular architecture, desmoplasia, areas of reduced cell density, and the presence of dense collagenous stroma surrounding the nodules, the tumor was classified as desmoplastic/nodular MB (**Supplementary Figure S1**).

A panel of three assays, beta-catenin, filamin A, and GAB1 staining, originally proposed by David Ellison et al., was used to immunohistochemically (IHC) confirm the diagnosis of MB and identify its genetically defined subtype (2, 15). Ki-67 was assessed as a marker of proliferation activity. An IHC study showed cytoplasmic expression of beta-catenin (**Figure 2D**), filamin A expression (**Figure 2E**), and GAB1 expression (**Figure 2F**). The level of Ki-67 in the tumor tissue registers at 25%–30% (**Supplementary Figure S2**).

Based on the findings from the histomorphological and histogenetic classification, the tumor was diagnosed as desmoplastic/nodular SHH-MB, Grade 4.

### Methods

Parents of the patient provided informed consent for molecular genetic testing. Approval for the study was granted by the institutional ethics committee under Protocol #3502-22 dated 21 February 2020.

The genomic DNA from peripheral blood and tumor samples were prepared for sequencing utilizing Kapa Biosystems (Roche) kits. To target the coding regions of the genome, the TruSeq Exome Capture kit (Illumina) was employed. The Fragment Analyzer ensured the quality of the resultant libraries, while qPCR assessed the adequacy of DNA quantity. Following quality assurance and quantification of DNA, the library pool was sequenced across two lanes of the Illumina NovaSeq 6000.

The raw sequencing data, presented as FASTQ files, were acquired through the utilization of bcl2fastq v2.20 Conversion Software (Illumina). Subsequently, both germline and somatic variant calling were executed following the best practices recommended by GATK and Mutect2, respectively (16, 17). Germline variants were independently found using two different variant calling tools—HaplotypeCaller (16) and DeepVariant (18), each run with default settings.

DeepVariant and HaplotypeCaller are advanced tools used to identify germline genetic variants from sequencing data. DeepVariant, developed by Google, uses deep learning techniques to achieve high accuracy in variant detection by converting raw sequence data into a list of genetic variants. HaplotypeCaller, part of the Broad Institute’s GATK toolkit, was considered the gold standard in the field. It builds possible haplotypes in a region and assigns probabilities to them to determine the most probable genetic variants.

When using “default settings”, these tools apply preset parameters optimized for common use cases. It is expected that the majority of routine research will use the default settings.

## Results

Initially, we sought to analyze somatic and germline DNA sequencing since the patient was presenting multiple severe phenotypes.

Initial analysis of somatic mutations indicated the presence of nine variants that passed the technical quality filtering: (1) six with non-coding annotation and (2) two long InDels that lacked convincing support upon examining the IGV view of the sequencing reads. The remaining missense variant in *SATL1* did not affect any conserved regions of the gene (MPC < 1). Given that *SATL1* is not an oncogene, this variant was dismissed as a diagnostic candidate.

Then, two relevant germline variants were identified—NC_000018.9:g.22805738T>C (NM_015461:p.Asp715Gly, rs1390185292) in the *ZNF521* and NC_000007.13:g.15197085856T>A (NM_170606:p.Thr316Ser, rs10454320) in the *KMT2C*. Both variants were assessed as “variants of uncertain significance” according to the joint recommendations of Clinical Genome Resource (ClinGen), Cancer Genomics Consortium (CGC), and Variant Interpretation for Cancer Consortium (VICC) (19).

For further variant interpretation, we investigated whether *ZNF521* has any prior evidence of being relevant to MB or other phenotypes. We used both pre-assembled lists of genes for screening (20) and the pathway analysis tools, such as WikiPathways (21) or Genepanel.iobio (22). Although *ZNF521* is a diagnostic gene for MB, it was absent from all lists used (23). *ZNF521* was previously shown to modulate the SHH pathway through binding to GLI1 and GLI2 (24). The rs1390185292 missense variant affects a highly conserved region of *ZNF521* (the Missense badness, PolyPhen-2, and Constraint summary metric score; MPC > 2.5) (25), potentially carrying serious implications for normal protein function. Further testing of rs1390185292 for *de novo* status in the patient revealed this SNP in one of the parents by Sanger sequencing data, preventing this variant from being considered as molecularly diagnostic, but leaving the possibility for incomplete penetrance effects.

According to the WGS study on relatively large MB cohorts, *KMT2C* is characterized by mutations of varying severity (26). The rs10454320 affects the highly conserved ePHD1_KMT2C domain “Extended PHD finger 1 found in histone-lysine N-methyltransferase 2C”, which is consistent with high levels of deleteriousness metrics (MPC>2.5) (25). This explains the high statistical difference in survival prognosis (27).

**Figure 3:**
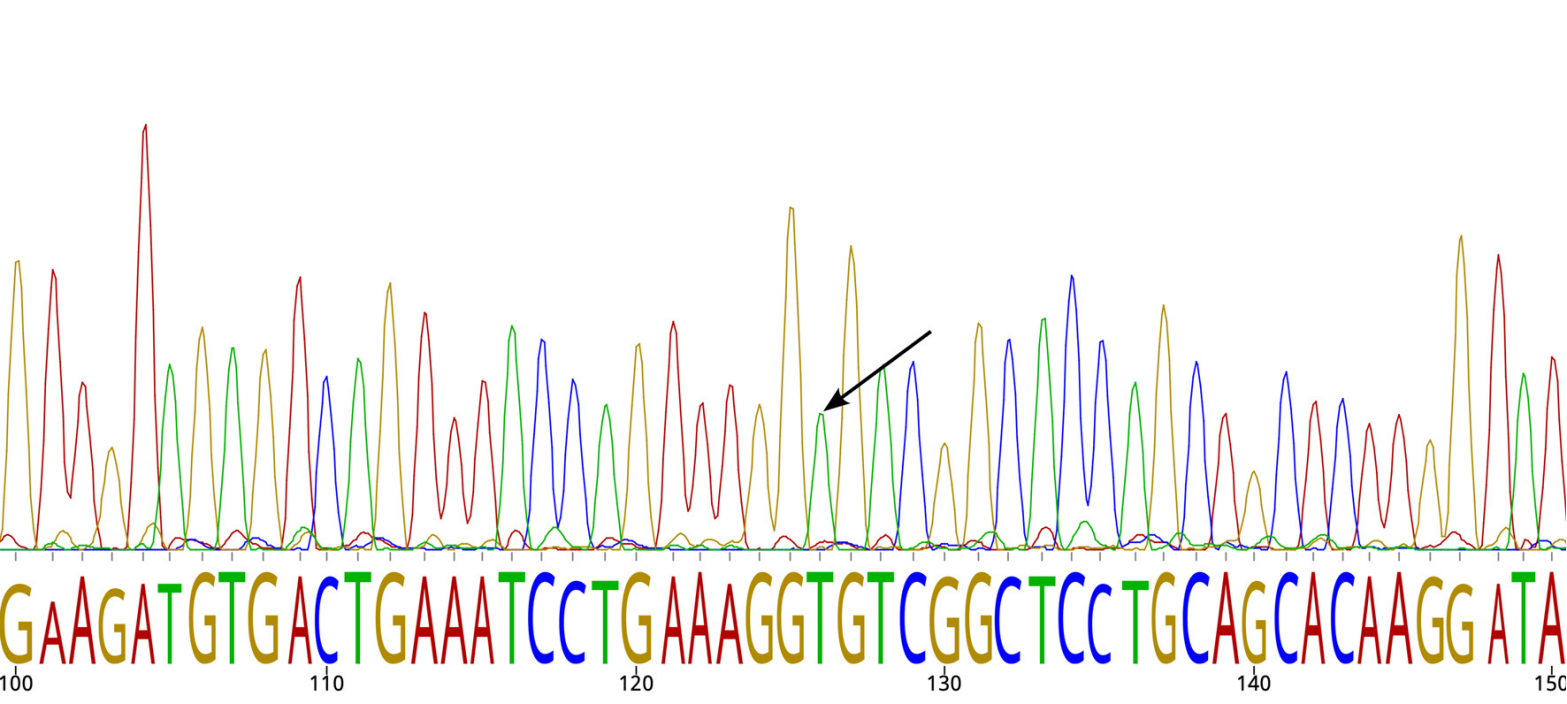
Sanger sequencing chromatogram. The arrow indicates the reference allele for SNP rs10454320.

The variant rs10454320 was filtered out by GATK’s best practice protocol for germline variants using HaplotypeCaller but was successfully identified by DeepVariant with default parameters. rs10454320 was filtered out by GATK’s best practice protocol for germline variants based on HaplotypeCaller (16), but was successfully identified by DeepVariant with default parameters (18). The variant rs10454320 was a false positive according to Sanger sequencing (**Figure 3**). The observation implies that simply comparing variant calling tools using established benchmarks is inadequate to promptly revise the current standards of clinical practice.

## Discussion

Clinical practice recommends tumor sequencing testing, but a fraction of patients affected by MB remain undiagnosed. Thus, the study of hereditary predisposition may provide additional insight into the contribution of rare/*de novo* variants to disease progression. However, germline analysis requires careful standardization of detection techniques specifically in the context of MB.

The false-positive rs10454320 in the *KMT2C* gene appears to be the result of a software problem rather than a technical error in the Illumina sequencer. Specifically, this problem was observed in DeepVariant but not in HaplotypeCaller, even though the data are the same.

The superiority of DeepVariant over other existing variant calling protocols based on synthetic parameters like precision and recall is known from the literature (28, 29). In some studies, it was shown that DeepVariant surpasses HaplotypeCaller in Mendelian error rate, Ti/Tv ratio, and clinical variant detection, although it faces challenges with quality score correlation and integration into downstream applications. The concordance rate between the two pipelines was 88.73%, indicating a high level of agreement in variant calling results. According to various articles, in summary, the difference between the protocols is attributed to the higher number of true-positive variants identified by DeepVariant (30). However, it has not yet been shown that choosing one or the other tool can dramatically determine the severity of the diagnosis and that sometimes this choice is not in favor of DeepVariant.

Adherence to standards of clinical practice is important not only for patient safety but also for maintaining the integrity and credibility of medical research. Without adherence to established standards, it becomes difficult to compare and replicate research findings, hindering the progress of medical science. Before integrating new tools into clinical practice, they must undergo a rigorous evaluation process to ensure their safety, efficacy, and practicality. This evaluation should consider established benchmarks and guidelines, as well as input from various constituents, including patients, clinicians, researchers, and regulatory authorities.

## Data Availability

The datasets presented in this study can be found in online repositories. The names of the repository/repositories and accession number(s) can be found below: https://www.ncbi.nlm.nih.gov/snp/, rs1390185292, https://www.ncbi.nlm.nih.gov/snp/, rs10454320.
