# Supplementary material for: Case report: A case study of variant calling pipeline selection effect on the molecular diagnostics outcome
Source: Front Oncol. 2024 Oct 31;14:1422811. doi: 10.3389/fonc.2024.1422811 (PMC11560904; doi:10.3389/fonc.2024.1422811)
Supplement: Supplementary file 1 [file DataSheet1.docx]

**
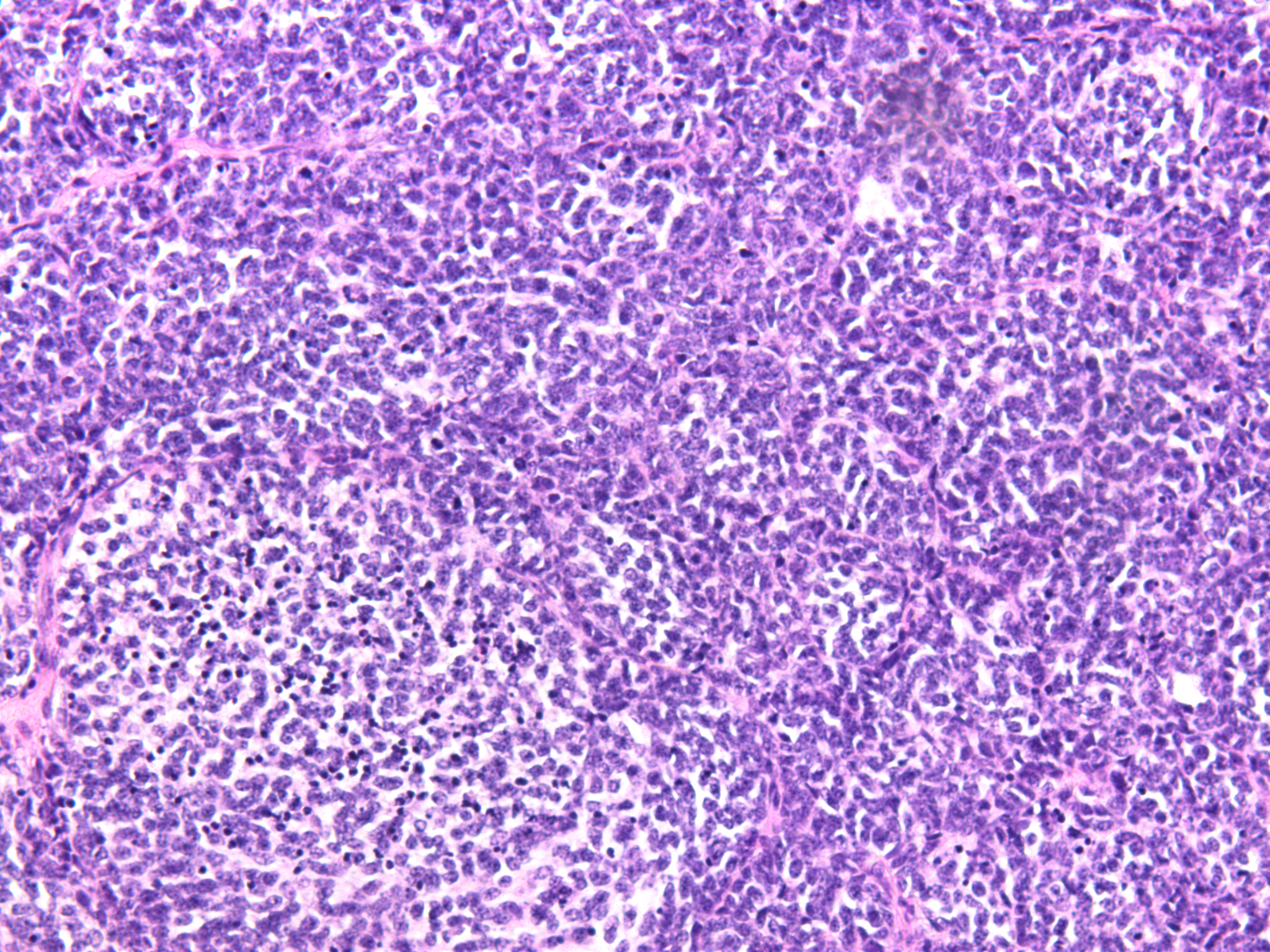
**

**Figure S1.** Hematoxylin-eosin staining of a tumor sample, magnified at ×200.

**
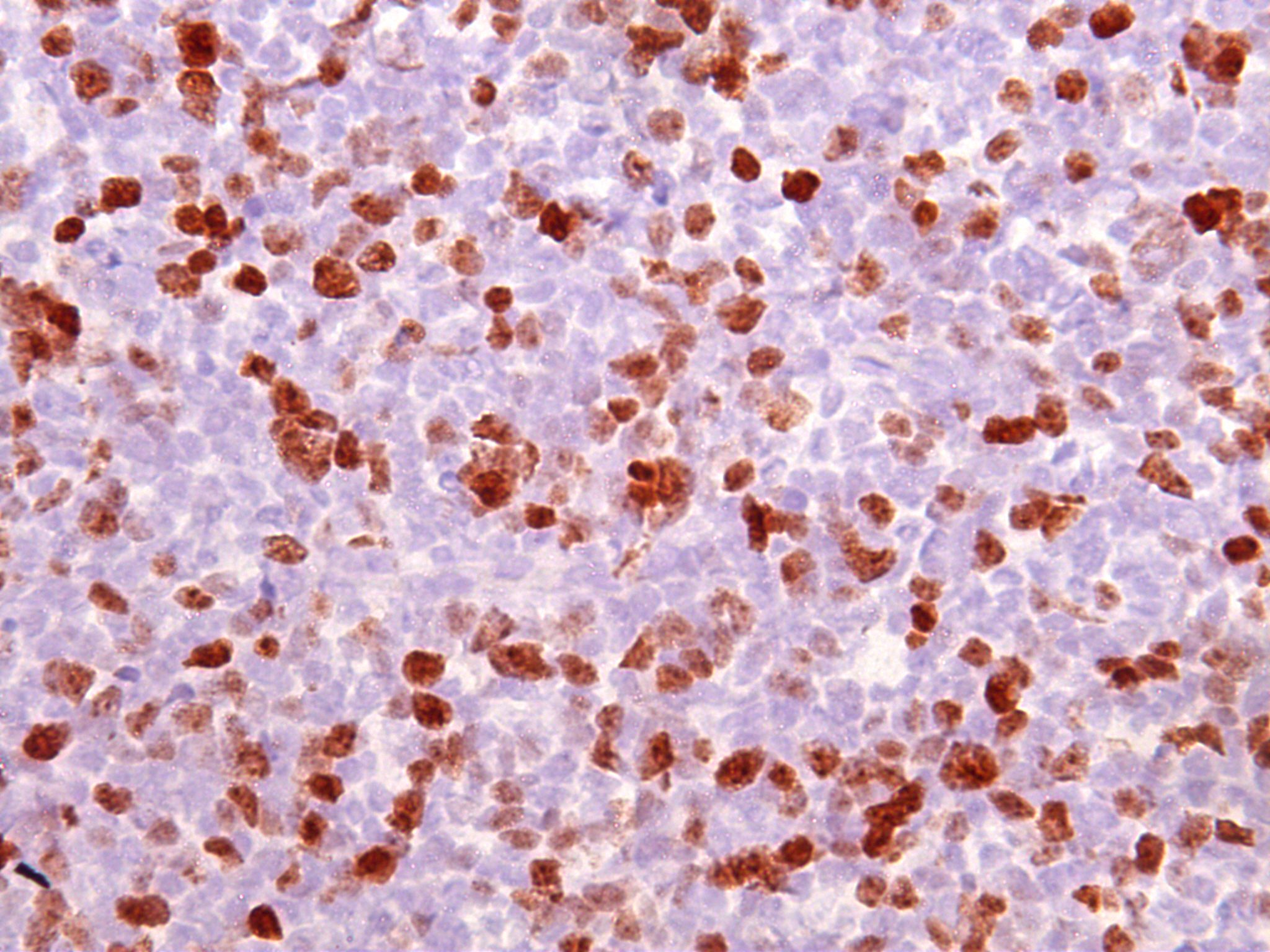
**

**Figure S2.** Ki-67 staining of a tumor sample, magnified at ×400.
